# Prevalence and control of hypertension among people living with HIV receiving care at a Nigerian hospital

**DOI:** 10.11604/pamj.2022.41.153.21809

**Published:** 2022-02-21

**Authors:** Idongesit Linus Jackson, Silas Monday Lawrence, Chioma Nneoma Igwe, Chinwe Victoria Ukwe, Matthew Jegbefume Okonta

**Affiliations:** 1 Department of Clinical Pharmacy and Biopharmacy, University of Uyo, Akwa Ibom State, Nigeria,; 2 College of Health Sciences, Etinan, Akwa Ibom State, Nigeria,; 3 Department of Clinical Pharmacy and Pharmacy Management, University of Nigeria, Nsukka, Enugu State, Nigeria

**Keywords:** Antiretroviral therapy, comorbidity, control, HIV, hypertension, prevalence

## Abstract

**Introduction:**

with the introduction of newer and safer antiretroviral drugs, HIV positive persons are now living longer. Consequently, cardiovascular diseases associated with ageing and chronic low grade inflammation due to the presence of the virus are increasingly found in this population. This study aimed to assess the prevalence and control of hypertension among people living with HIV (PLHIV) receiving care at a Nigerian hospital.

**Methods:**

this cross-sectional study was conducted as part of the Patient´s HIV Knowledge Questionnaire (PHKQ) validation study among HIV positive outpatients at the University of Uyo Teaching Hospital, Uyo, Akwa Ibom State, Nigeria. Hypertension was self-reported and confirmed by a documented physician diagnosis in the patient´s case notes and/or self-reported use of antihypertensive medication. For each participant, the average of two close blood pressure (BP) measurements obtained using an automatic upper arm BP monitor was taken as the BP. Hypertension control was defined as SBP <140 mmHg and DBP <90 mmHg. Data were analysed using the Statistical Product and Service Solutions (SPSS) v.21.0. Results were considered significant at p < 0.05.

**Results:**

prevalence of hypertension among PLHIV was 24.9%. Age (OR = 1.112, CI = 1.074 – 1.151, p < 0.001), body mass index (OR = 1.087, CI = 1.024 – 1.154, p = 0.004) and duration on antiretroviral therapy (OR = 1.169, CI = 1.090 – 1.254, p < 0.001) significantly predicted hypertension. Only 19 (24.4%) participants had controlled hypertension.

**Conclusion:**

hypertension is common among PLHIV seeking care at a Nigerian hospital. However, its control remains suboptimal. Regular screening for hypertension, its appropriate treatment and optimal control are essential in PLHIV.

## Introduction

Improved methods of detection of the human immunodeficiency virus (HIV) as well as the availability of more effective management modalities have helped in reducing morbidity and improving the survival of people living with HIV (PLHIV) [1]. This, in turn, has led to an increase in the long term complications of HIV infection, particularly associated with non-communicable diseases such as dyslipidemia, insulin resistance and hypertension [1-3].

Hypertension, the leading risk factor for cardiovascular and cerebrovascular mortality worldwide [4] seems to be common in HIV infected individuals [5-9], and continues to be on the increase [10]. Varying prevalence rates of hypertension among PLHIV have been reported: Kwarisiima *et al*. [11] reported a prevalence of 11% in rural Uganda; Baekken *et al*. [12] reported a prevalence of 31.7% in Norway; a South African study recorded a prevalence of 41.2% [13]. Results of a systematic review indicated an estimated global hypertension prevalence of 25.2% among PLHIV [14]. In Nigeria, prevalence rates range from 12.3% in Ido Ekiti, Ekiti State [15] to as high as 50.3% in Jos, Plateau state [5].

The presence of established risk factors such as obesity, diabetes mellitus and renal insufficiency [16], as well as HIV-related factors have been adduced to explain the increasing incidence and prevalence of hypertension in PLHIV [16-19]. Continuous activation of the immune system and persistent inflammation, which are common in HIV infection, are implicated in the development of cardiovascular disease in this population [20]. Also, antiretroviral drugs (ARDs) such as protease inhibitors (e.g. lopinavir/ritonavir) and zidovudine-lamivudine-nevirapine ARD combination have been associated with the development of hypertension in PLHIV [5, 17, 21]. Isa *et al*. [5] reported that 31% of PLHIV in their study setting developed hypertension 12 months after initiating antiretroviral therapy (ART). However, there are contrasting evidence that the use of ARDs appears to lower the prevalence of hypertension [12], or not even associated with hypertension [9, 15, 22]. Yet, there are studies which have reported higher prevalence in HIV-negative adults compared with HIV-positive adults [11, 23, 24]. Regardless of the relationship between both conditions, it has been argued that countries with a high burden of HIV like Nigeria may also have a high burden of non-communicable diseases, e.g., hypertension [25].

The higher the blood pressure (BP), the greater the likelihood of developing heart attack, heart failure, stroke, and kidney diseases. The ultimate public health goal of antihypertensive therapy, therefore, is to reduce cardiovascular and renal morbidity and mortality by treating systolic blood pressure (SBP) and diastolic blood pressure (DBP) to targets that are <140/90 mmHg [26, 27]. Nevertheless, studies [10, 21, 28] suggest that the management of hypertension remains unsatisfactory globally. A retrospective study conducted in Ugandan [10] for instance, reported that only 23% of HIV infected individuals with comorbid hypertension received antihypertensive treatment within the period under review; in the HIV-HY study [28], more than half of hypertensive PLHIV had uncontrolled hypertension. A study conducted among PLHIV in Brazil [29] indicated that only 20.9% of patients who were aware of their hypertensive condition were controlled. In a longitudinal cohort study of PLHIV in Oslo [30], hypertension control was obtained in only 22% of patients on antihypertensive therapy. In another study [11], 85% of HIV-infected individuals who were hypertensive were not taking medication for hypertension, and half of those on medication had uncontrolled hypertension. Such studies are however scanty in Nigeria. Thus, the present study was conducted to assess the prevalence and control of hypertension among PLHIV receiving care at a Nigerian tertiary health institution.

## Methods

**Study population and data collection:** this was a cross-sectional study conducted as part of the Patient´s HIV Knowledge Questionnaire (PHKQ) validation study [31] among HIV positive persons attending clinic at the University of Uyo Teaching Hospital (UUTH). UUTH is a 500-bed capacity, government-owned, tertiary hospital located in Uyo, the capital city of Akwa Ibom State, Nigeria. At the time of data collection, HIV care services were being supported by the Family Health International (FHI) - 360 in collaboration with the United States Agency for International Development (USAID). Sample size determination had been described in the validation study [31]. The study included adult (at least 18 years) HIV positive persons who had been on ART for at least 3 months, and who gave informed consent to participate. Patients with cognitive impairment or who were too ill to participate were excluded. Participants were recruited by convenience. Hypertension was self-reported as a response to the question: ‘Do you have high blood pressure?´ An affirmative answer to this question was confirmed by physician diagnosis documented in the patient´s case notes and/or self-reported use of antihypertensive medication.

Blood pressure measurements were taken at the clinic by trained staff using a validated automatic upper arm BP monitor (Omron M3, Omron Healthcare Co., Ltd. Kyoto, Japan). Three readings were measured 30 seconds apart on the left upper arm, with the participant being still in a sitting position following at least 10 minutes of rest. The average of two close readings was taken as the BP. Hypertension control was defined as SBP <140 mmHg and DBP <90 mmHg. We used a goal of <140/90 mmHg due to the presence of comorbid HIV infection, and the increased susceptibility to metabolic abnormalities and kidney disease in PLHIV. This is in line with the Eight Report of the Joint National Committee on Detection, Evaluation, and Treatment of High BP (JNC 8) which recommends a goal BP of <140/90 mmHg in adults with diabetes or chronic kidney disease [27]. Weight and height were measured as part of the clinic routine and documented in the case notes. These were used in the determination of the body mass index (BMI), defined as weight in kilograms divided by height in meters squared, for each participant. The study was conducted between August and September 2018.

**Ethics approval:** approval to conduct this study was obtained from the Health Research Ethical Committee of UUTH (approval number: UUTH/AD/S/96/VOLXXI/188). Written informed consent was obtained from participants.

**Statistical analysis:** data were coded and analysed using the Statistical Product and Service Solutions (SPSS, IBM Inc, Chicago, IL) v. 21.0. Frequencies and percentages were used to summarize categorical data; mean (standard deviation, SD) and median (interquartile range, IQR) were used to summarize continuous data with normal and non-normal distributions, respectively. Binary logistic regression analyses were carried out to examine associations between each patient variable and hypertension. However, length of time since HIV diagnosis was not used in the analyses due to its collinearity with duration on ART. Finally, the significant variables were included in a multivariate logistic regression model to determine the true predictors of hypertension. Missing data were handled by list wise deletion. All analyses were considered significant at p < 0.05.

## Results

Out of the 430 eligible patients approached, 417 agreed to participate, giving a response rate of 97.0%. Those who declined gave reasons for non-participation such as lack of interest, lack of time, or inability to read. Females constituted the majority (68.6%) of our study population. The median (IQR) age was 38 (32 - 47) years. Most (44.1%) of the respondents had had up to secondary education. About half of them were married (56.4%) and currently employed (53.2%) (Table 1).

**Table 1:** socio-demographic characteristics of respondents (N=417)

Characteristic	Frequency	Percentage
**Gender**		
Male	131	31.4
Female	286	68.6
Education*		
None	7	1.7
Primary	46	11.1
Secondary	184	44.3
Tertiary	178	42.9
**Marital Status**		
Single	119	28.5
Married	235	56.4
Divorced/Widowed	63	15.1
**Employment Status**		
Working	222	53.2
Unemployed	143	34.3
Retired	23	5.5
Student	29	7.0
**BMI** (Kg/m^2^)**		
<18.5	11	2.8
18.5 – 24.9	214	53.6
25.0 – 29.9	118	29.6
≥30.0	56	14.0
	**Median**	**IQR**
**Age** (years)	38	32 – 47
**Length of time since HIV Diagnosis** (years)	8	4 – 11
**Duration on ART** (years)	7	3 – 11

* N = 415; **N = 399; SD – standard deviation; IQR – interquartile range; ART – antiretroviral therapy.

Prevalence of hypertension among our study population was 24.9%. Binary logistic regression indicated that age, body mass index (BMI), marital status and duration on ART were independent predictors of hypertension. However, marital status was not a significant predictor in the final regression model. Increasing age (odds ratio, OR = 1.112; confidence interval, CI = 1.074 - 1.151; p < 0.001), BMI (OR = 1.087; CI = 1.024 - 1.154; p = 0.004) and duration of treatment with ARDs (OR = 1.169; CI = 1.090 - 1.254; p < 0.001) were associated with increased odds of developing hypertension (Table 2).

**Table 2:** results of binary logistic regression to determine predictors of hypertension

Variable	Unadjusted OR (CI)	P	Adjusted OR (CI)	P
**Gender** (male)	1.441 (0.905 – 2.296)	NS	-	-
**Age (years)**	1.147 (1.112 – 1.182)	0.000	1.112 (1.074 – 1.151)	0.000
**Employment status** (unemployed)	1.019 (0.653 – 1.589)	NS	-	-
**Marital status**				
Single	-	0.000	-	NS
Married	4.080 (2.009 – 8.285)	0.000	-	NS
Divorced/widowed	9.909 (4.387 – 22.381)	0.000	-	NS
**Duration on ART**	1.262 (1.186 – 1.344)	0.000	1.169 (1.090 – 1.254)	0.000
**Education**				
None	-	NS	-	-
Primary	0.786 (0.133 – 4.633)	NS	-	-
Secondary	0.933 (0.175 – 4.964)	NS	-	-
Tertiary	0.748 (0.140 – 4.000)	NS	-	-
BMI (Kg/m^2^)	1.074 (1.023 – 1.129)	0.004	1.087 (1.024 – 1.154)	0.006

BMI = Body mass index; CI= confidence interval; NS = Not significant; OR = odds ratio; Correct predictions = 80.7%, Nalgelkerke R^2^ = 42.5%, **χ^2^**= 135.991, df = 5, p < 0.001.

Majority (75.6%) of our hypertensive participants had uncontrolled hypertension; only 19 (24.4%) were controlled. Eighty-four (83.2%) were already on ART before hypertension was diagnosed; only 8 (7.9%) reported prior knowledge of their hypertension diagnosis before ART was commenced. Nine (8.9%) were diagnosed with hypertension about the same time they tested positive for HIV.

## Discussion

The present study aimed to determine the prevalence and control of hypertension among PLHIV seeking care at a tertiary health facility in Nigeria. We found a hypertension prevalence of 24.9% among the study population. Mean SBP and DBP were 152 ± 20 mmHg and 90 ± 11 mmHg respectively. Patient age, BMI, and length of time on ART were significant predictors of hypertension in the multivariate logistic regression model. Hypertension control was observed only in 24.4% of hypertensive participants. Hypertension prevalence in our study is similar to findings of earlier studies [14, 29]. However, this value is much higher than the prevalence reported in Cross River State, Nigeria [32], and in Uganda [11] but lower than results of studies conducted in Northern Nigeria [5], New York City [33] and Malaysia [22].

Our study showed that only about a quarter of hypertensive patients had controlled hypertension. This is similar to reports of previous studies [28, 30], but lower than the control rates observed in earlier studies [11, 28, 33]. A Tanzanian study [8] however reported a much lower control of 2.3% among HIV-infected individuals studied. This could have been due to the fact that only about a quarter of the patients in that study knew that they were hypertensive, and only 16.3% of the participants were on antihypertensive drugs. In our study, however, hypertension was self-reported (and confirmed by physician diagnosis documented in the case notes). Hence, participants were already aware of their hypertension diagnosis and had been on antihypertensive medications prior to the study. Our study thus agree with results of a systematic review which indicated that the rates of hypertension control in the general African population is low, never exceeding 45% irrespective of levels of awareness and treatment [34].

Poor BP control, both in HIV-infected and in the general uninfected population, can be due to a variety of causes including poor adherence on the part of the patient (which may be due to a myriad of reasons), costs of medication, clinical inertia, poor patient-provider communication, interactions of antihypertensive medications with food, herbs or other drugs, and even psychological factors. Nevertheless, efforts to manage hypertension adequately in people infected with HIV should be intensified to reduce the risk of cardiovascular disease, which is increasingly associated with mortality in this population [35].

In line with earlier studies [5, 6, 11, 22], patient age significantly predicted hypertension in our study. A similar Nigerian study [32] found an association between age and hypertension in bivariate analysis, but this was not significant in the multivariate logistic regression. Duration on ART was the strongest predictor of hypertension in this study. Increasing duration on ART was associated with increased odds of developing hypertension. Thus, for every additional year on ART, the odds of developing hypertension increases by 17% adjusting for age, BMI and marital status, which were independent predictors of hypertension in our study participants. Our result corroborates the report from a previous Nigerian study [5] in which hypertension was found to have developed in a further 31% of the studied cohorts 12 months after commencing ART. Though a Tanzanian study [8] did not find an association between hypertension and duration on ART, HIV-positive individuals who had been on ART for more than two years were found to have a two-fold odds of developing hypertension than HIV-negative controls, even after adjusting for age and gender. In their study, Arruda *et al*. [6] observed that duration on ART independently predicted hypertension, though it was not a significant predictor in the final logistic model.

The result of the present study is in line with reports of earlier studies [5, 9, 11, 15, 22] which indicated that BMI is a predictor of hypertension in PLHIV. Hence, our result buttresses the fact that BMI is an established risk factor for developing hypertension both in the general population and in HIV-infected individuals [11, 16, 19]. More than three-quarters of our respondents who were hypertensive were already on ART before the diagnosis of hypertension was made. This finding may be attributed at least in part, to the use of ART such as; zidovudine-lamivudine-nevirapine which has been implicated in the development of hypertension [21]. Though this was not within the scope of the present research, this drug combination (zidovudine-lamivudine-nevirapine) was the predominant ARD combination that was being prescribed at the time of data collection in the study setting. Development of hypertension after a period of time on ART has been previously documented [5, 31].

**Limitations:** the results of our study should be interpreted in the light of some limitations: the use of a single health facility might limit the generalizability of our study results. Further, the cross-sectional study design made it impossible for causality to be ascertained. Also, clinical outcomes (such as viral load and cluster of differentiation-4 [CD4]) and lifestyle such as cigarette smoking, alcohol intake, diet, exercise, etc were not assessed. Thus, we could not examine the possible (confounding) effects of these on the prevalence and control of hypertension. Most importantly, BP was not measured for every patient, and among those who reported being hypertensive, measurements were available for only seventy-five percent, partly because the primary aim of the study was the validation of the PHKQ [31]. Therefore, an absolute conclusion with respect to the prevalence of hypertension and its control could not be made.

## Conclusion

Hypertension seems to be common in PLHIV probably due to traditional risk factors and HIV-related factors. However, its control in this population remains suboptimal. There is a need for collaborative healthcare professional team work to assess and address possible factors responsible for suboptimal hypertension control in hypertensive PLHIV.

### What is known about this topic

- Hypertension is common among people living with HIV;
- Traditional risk factors as well as HIV-related factors are probable causes of hypertension in this population.

### What this study adds

- Most cases of hypertension in HIV positive persons are diagnosed after initiating antiretroviral therapy;
- Despite the knowledge of hypertension diagnosis, control of hypertension in people living with HIV is suboptimal;
- It should be noted, however, that these findings are limited to our study setting.

## References

[1] Young F, Critchley JA, Johnstone LK, Unwin NC (2009). A review of co-morbidity between infectious and chronic disease in Sub Saharan Africa: TB and diabetes mellitus, HIV and metabolic syndrome, and the impact of globalization. Global Health.

[2] Petersen M, Yiannoutsos CT, Justice A, Egger M (2014). Observational research on NCDs in HIV-positive populations: conceptual and methodological considerations. J Acquir Immune Defic Syndr.

[3] Hadigan C, Meigs JB, Corcoran C, Rietschel P, Piecuch S, Basgoz N (2001). Metabolic abnormalities and cardiovascular disease risk factors in adults with human immunodeficiency virus infection and lipodystrophy. Clin Infect Dis.

[4] World Health Organization (2009). Global Health Risks: Mortality and burden of disease attributable to selected major risks. The World Health Organization.

[5] Isa SE, Kang´ombe AR, Simji GS, Shehu NY, Oche AO, Idoko JA (2017). Hypertension in treated and untreated patients with HIV?: A study from 2011 to 2013 at the Jos University Teaching Hospital, Nigeria. Trans R Soc Trop Med Hyg.

[6] de Arruda Junior ER, Lacerda HR, Moura LCRV, de Fatima Pessoa Militão de Albuquerque M, de Barros Miranda Filho D (2010). Risk factors related to hypertension among patients in a cohort living with HIV/AIDS. Braz J Infect Dis.

[7] Gazzaruso C, Bruno R, Garzaniti A, Giordanetti S, Fratino P, Sacchi P (2003). Hypertension among HIV patients: Prevalence and relationships to insulin resistance and metabolic syndrome. J Hypertens.

[8] Peck RN, Shedafa R, Kalluvya S, Downs JA, Todd J, Suthanthiran M (2014). Hypertension, kidney disease, HIV and antiretroviral therapy among Tanzanian adults: A cross-sectional study. BMC Med.

[9] Medina-Torne S, Ganesan A, Barahona I, Crum-Cianflone NF (2012). Hypertension is common among HIV-infected persons, but not associated with HAART. J Int Assoc Physicians AIDS Care (Chic).

[10] Kalyesubula R, Kayongo A, Semitala FC, Muhanguzi A, Katantazi N, Ayers D (2016). Trends and level of control of hypertension among adults attending an ambulatory HIV clinic in Kampala, Uganda: a retrospective study. BMJ Glob Health.

[11] Kwarisiima D, Balzer L, Heller D, Kotwani P, Chamie G, Clark T (2016). Population-Based Assessment of Hypertension Epidemiology and Risk Factors among HIV-Positive and General Populations in Rural Uganda. PLoS One.

[12] Baekken M, Os I, Sandvik L, Oektedalen O (2008). Hypertension in an urban HIV-positive population compared with the general population: influence of combination antiretroviral therapy. J Hypertens.

[13] Mutemwa M, Peer N, de Villiers A, Mukasa B, Matsha TE, Mills E (2018). Prevalence, detection, treatment, and control of hypertension in human immunodeficiency virus (HIV)-infected patients attending HIV clinics in the Western Cape Province, South Africa. Medicine (Baltimore).

[14] Xu Y, Chen X, Wang K (2017). Global prevalence of hypertension among people living with HIV: A systematic review and meta-analysis. J Am Soc Hypertens.

[15] Ogunmola OJ, Oladosu OY, Olamoyegun AM (2014). Association of hypertension and obesity with HIV and antiretroviral therapy in a rural tertiary health center in Nigeria: A cross-sectional cohort study. Vasc Health Risk Manag.

[16] Okeke NL, Davy T, Eron JJ, Napravnik S (2016). Hypertension Among HIV-infected Patients in Clinical Care 1996-2013. Clin Infect Dis.

[17] Muhammad S, Sani MU, Okeahialam BN (2013). Cardiovascular disease risk factors among HIV-infected Nigerians receiving highly active antiretroviral therapy. Niger Med J.

[18] Justice AC (2006). Prioritizing primary care in HIV: comorbidity, toxicity, and demography. Top HIV Med.

[19] Rodriguez-Arboli E, Mwamelo K, Kalinjuma AV, Furrer H, Hatz C, Tanner M (2017). Incidence and risk factors for hypertension among HIV patients in rural Tanzania: A prospective cohort study. PLoS One.

[20] Ipp H, Zemlin A (2013). The paradox of the immune response in HIV infection: When inflammation becomes harmful. Clin Chim Acta.

[21] Divala OH, Amberbir A, Ismail Z, Beyene T, Garone D, Pfaff C (2016). The burden of hypertension, diabetes mellitus, and cardiovascular risk factors among adult Malawians in HIV care: consequences for integrated services. BMC Public Health.

[22] Hejazi N, Huang MSL, Lin KG, Choong LCK (2013). Hypertension among HIV-infected adults receiving highly active antiretroviral therapy (HAART) in Malaysia. Glob J Health Sci.

[23] Dillon DG, Gurdasani D, Riha J, Ekoru K, Asiki G, Mayanja BN (2013). Association of HIV and ART with cardiometabolic traits in sub-Saharan Africa: a systematic review and meta-analysis. Int J Epidemiol.

[24] Malaza A, Mossong J, Bärnighausen T, Newell M-L (2012). Hypertension and obesity in adults living in a high HIV prevalence rural area in South Africa. PLoS One.

[25] Angkurawaranon C, Nitsch D, Larke N, Rehman A, Smeeth L, Addo J (2016). Ecological Study of HIV Infection and Hypertension in Sub-Saharan Africa: Is There a Double Burden of Disease?. PLoS ONE.

[26] Hansson L, Zanchetti A, Carruthers SG, Dahlöf B, Elmfeldt D, Julius S (1998). Effects of intensive blood-pressure lowering and low-dose aspirin in patients with hypertension: Principal results of the Hypertension Optimal Treatment (HOT) randomized trial. HOT Study Group. Lancet.

[27] James PA, Oparil S, Carter BL, Cushman WC, Dennison-Himmelfarb C, Handler J (2014). 2014 Evidence-Based guideline for the management of high blood pressure in adults: Report from the panel members appointed to the eighth Joint National Committee (JNC 8). JAMA.

[28] De Socio G, Ricci E, Maggi P, Parruti G, Pucci G, Di Biagio A (2014). Prevalence, Awareness, Treatment, and Control Rate of Hypertension in HIV-Infected Patients: The HIV-HY Study. Am J Hypertens.

[29] Evanizio A, Heloisa L, MouraLíbia AM, de Barros FD, George D (2010). Profile of patients with Hypertension Included in a Cohort with HIV/ AIDS in the State of Pernambuco, Brazil. Arq Bras Cardiol.

[30] Manner IW, Baekken M, Oektedalen O, Os I (2012). Hypertension and antihypertensive treatment in HIV-infected individuals. A longitudinal cohort study. Blood Pressure.

[31] Jackson IL, Okonta JM, Ukwe CV (2020). Development and psychometric evaluation of the Patient´s HIV Knowledge Questionnaire (PHKQ). Int J Clin Pharm.

[32] Okpa H, Bisong E, Enang O, Monjok E, Essien E (2017). Predictors of hypertension in an urban HIV-infected population at the University of Calabar Teaching Hospital, Calabar, Nigeria. HIV AIDS (Auckl).

[33] Merle M, Eduard P, Ehrin A, Shari K, Victoria S, Heejung B (2014). Prevalence, Treatment, and Control of Dyslipidemia and Hypertension in 4278 HIV Outpatients. J Acquir Immune Defic Syndr.

[34] Kayima J, Wanyenze RK, Katamba A, Leontsini E, Nuwaha F (2013). Hypertension awareness, treatment and control in Africa: a systematic review. BMC Cardiovasc Disord.

[35] Frank Jr P, Rose B, Anne M, Joan C, Kathleen W, John B (2006). Mortality in the Highly Active Antiretroviral Therapy Era: Changing causes of death and disease in the HIV Outpatient Study. J Acquir Immune Defic Syndr.

